# Advancing the Diagnosis and Treatment of Early Chronic Pancreatitis Through Innovation in Imaging and Biomarker Profiling—A Narrative Review

**DOI:** 10.3390/life15101574

**Published:** 2025-10-09

**Authors:** Alexandru-Ionut Coseru, Diana Elena Floria, Constantin Simiras, Radu Alexandru Vulpoi, Vadim Rosca, Roxana Nemteanu, Oana Petrea, Irina Ciortescu, Oana-Bogdana Barboi, Gheorghe G. Balan, Catalin Sfarti, Georgiana-Emanuela Gîlca-Blanariu, Catalina Mihai, Liliana Gheorghe, Alina Plesa, Vasile-Liviu Drug

**Affiliations:** 1 Department of Gastroenterology, Grigore T. Popa University of Medicine and Pharmacy, 700115 Iasi, Romania; ionutz_ionutz_barlad@yahoo.com (A.-I.C.); vroshca94@gmail.com (V.R.); maxim_roxxana@yahoo.com (R.N.); stoicaoanacristina28@gmail.com (O.P.); irinaciortescu@yahoo.com (I.C.); oany_leo@yahoo.com (O.-B.B.); balan.gheo@me.com (G.G.B.); cvsfarti@gmail.com (C.S.); catalinamihai@yahoo.com (C.M.); liliana.gheorghe@umfiasi.ro (L.G.); alinaplesaro@yahoo.com (A.P.); vasidrug@email.com (V.-L.D.); 2 Institute of Gastroenterology and Hepatology, “St. Spiridon” University Hospital, 700111 Iasi, Romania; c_simiras@yahoo.com; 3 Department of Radiology, “Saint. Spiridon” Hospital, 700111 Iasi, Romania

**Keywords:** early chronic pancreatitis, biomarkers, chronic pancreatitis, endoscopic ultrasound, recurrent acute pancreatitis, therapeutic endoscopy, pancreatic function, Rosemont criteria, early parenchymal changes

## Abstract

Early chronic pancreatitis (ECP) represents a potentially reversible stage in the natural history of chronic pancreatic disease. Timely diagnosis of ECP offers a possibility for intervention, yet its diagnosis remains challenging due to nonspecific symptoms, lack of standardized criteria, and the limited diagnostic sensitivity of conventional tools. This review aims to synthesize recent advancements in the understanding, detection, and management of ECP, with a focus on innovation in imaging techniques and biomarker profiling. The goal is to facilitate earlier diagnosis and more effective patient stratification. We reviewed the literature from the past five years, including original studies, meta-analyses, and expert consensus statements, to address the current evidence across genetic, inflammatory, imaging, and biochemical domains relevant to ECP. Endoscopic ultrasound and advanced magnetic resonance techniques offer high sensitivity in detecting early parenchymal changes, although inter-observer variability and lack of standardization persist. Biomarker discovery has focused on inflammatory (IL-6, sCD163), fibrotic (TGF-β1, TIMP-1), and oxidative markers, as well as novel candidates like microRNAs. Genetic predisposition (PRSS1, SPINK1, CTRC, CPA1, CLDN2) significantly influences disease onset and progression and could enable selection of high-risk individuals. Therefore, diagnosing ECP should involve a multidisciplinary precision-based approach integrating clinical, radiologic, molecular, serologic, and genetic data for individualized risk stratification.

## 1. Introduction

Chronic pancreatitis (CP) is an inflammatory condition characterized by progressive and irreversible destruction of pancreatic parenchyma, fibrosis, and loss of exocrine and endocrine function [1]. Clinical manifestations such as abdominal pain, steatorrhea, and diabetes are well recognized, yet the early stages of CP remain poorly understood and underdiagnosed [2].

The concept of early chronic pancreatitis (ECP) is defined as a distinct borderline clinical entity between acute or recurrent acute pancreatitis (RAP) and established CP [1,2,3]. Identifying ECP may provide an opportunity for timely intervention, as parenchymal changes may still be reversible and progression toward end-stage fibrosis has the potential to be discontinued [4,5,6].

**Figure 1:**
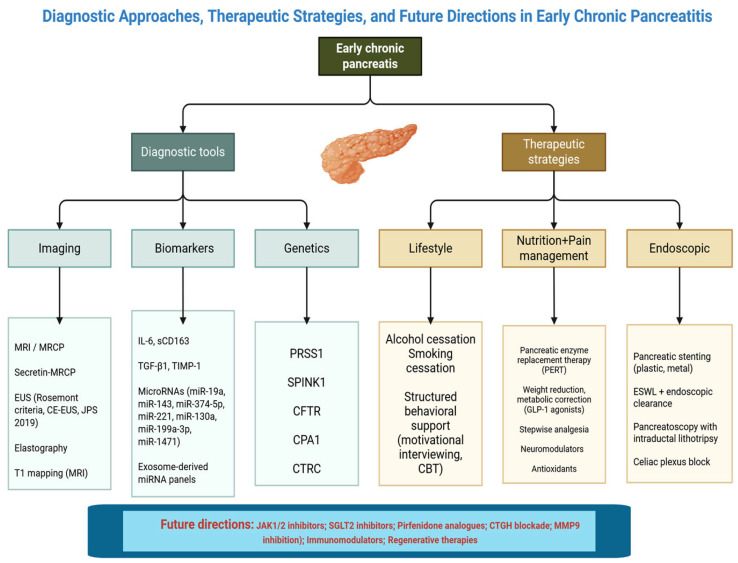
Diagnostic Approaches, Therapeutic Strategies, and Future Directions in Early Chronic Pancreatitis. Figure 1 provides a schematic overview of the current conceptual framework for early chronic pancreatitis (ECP). Diagnostic tools include imaging modalities such as magnetic resonance imaging/magnetic resonance cholangiopancreatography (MRI/MRCP), secretin-enhanced MRCP, endoscopic ultrasound (EUS) based on Rosemont criteria, contrast-enhanced EUS (CE-EUS) and elastography, as well as T1 mapping. Biomarkers investigated in ECP comprise inflammatory mediators (interleukin-6, soluble CD163, transforming growth factor-β1 [IL-6, sCD163, TGF-β1]), tissue inhibitors of metalloproteinases (TIMP-1), and circulating microRNAs (miR-19a, miR-143, miR-374-5p, miR-221, miR-130a, miR-199a-3p, miR-1471), including exosome-derived miRNA panels. Genetic predisposition is highlighted by mutations in PRSS1, SPINK1, CFTR, CPA1, and CTRC. Therapeutic strategies address lifestyle interventions (alcohol and smoking cessation, structured behavioural support such as cognitive behavioural therapy [CBT]), nutrition and pain management through pancreatic enzyme replacement therapy (PERT), metabolic correction with glucagon-like peptide-1 (GLP-1) agonists, stepwise analgesia, neuromodulators, and antioxidants. Endoscopic approaches include pancreatic stenting, extracorporeal shockwave lithotripsy (ESWL) with endoscopic clearance, pancreatoscopy with intraductal lithotripsy, and celiac plexus block. Future directions emphasize antifibrotic and immunomodulatory therapies such as JAK1/2 inhibitors, SGLT2 inhibitors, pirfenidone analogues, connective tissue growth factor (CTGF) blockade, and matrix metalloproteinase-9 (MMP9) inhibition, as well as regenerative strategies.

Figure 1 provides a schematic overview of the current conceptual framework for ECP.

Epidemiological data on ECP remain scarce. Population-based studies suggest that early disease is infrequent in the general population, but the risk is significantly higher among individuals with recurrent acute pancreatitis or established risk factors such as alcohol misuse and genetic predisposition. A European cohort estimated the overall prevalence of chronic pancreatitis at approximately 10 per 100,000 persons, with ECP accounting for only a minority of cases [7]. In a Dutch population-based study, the cumulative incidence of chronic pancreatitis after recurrent acute pancreatitis was 13% at 10 years, underscoring the importance of early recognition [8].

ECP challenges the notion that CP evolves as a continuum and irreversibly from RAP [9]. New data suggests that a subset of patients experience subclinical or minimally symptomatic disease that slowly progresses, influenced by complex interactions between genetic predisposition, immune dysregulation, lifestyle, and environmental triggers [8,9]. The absence of clear diagnostic criteria and no specific biomarkers may delay its diagnosis and stratification of patients at risk for disease progression. This knowledge gap highlights the need for validated tools and consensus frameworks to define ECP both radiologically and biologically [10,11].

The 2018 International Consensus Statements on Early Chronic Pancreatitis, led by Whitcomb et al., provided a conceptual model based on mechanistic disease pathways [12]. The 2019 revision of the Japanese Pancreas Society (JPS) guidelines marked new concepts, introducing a structured set of diagnostic criteria based on imaging, clinical, and functional parameters [13,14,15]. Nevertheless, these criteria remain underutilized in clinical practice, and their external validation is limited.

Imaging modalities such as high-resolution magnetic resonance imaging (MRI), endoscopic ultrasound (EUS), and elastography have significantly improved the detection of early parenchymal and ductal changes, particularly in patients without overt clinical symptoms [16]. Quantitative techniques like T1-weighted mapping and shear wave elastography provide objective metrics that may reflect subclinical fibrosis or inflammation, allowing for earlier detection and staging [16,17,18]. Advanced imaging has thus become a cornerstone in ECP research and diagnosis, yet remains limited by inter-operator variability, lack of standardization, and incomplete correlation with histopathologic changes [19,20,21].

Advanced imaging has thus become a cornerstone in ECP research and diagnosis, yet remains limited by inter-operator variability, lack of standardization, and incomplete correlation with histopathologic changes [19,20]. There is also increasing interest in molecular and serological markers that may complement imaging in the future [22,23]. This narrative review aims to critically appraise recent literature on advances in the diagnosis and management of ECP, with a particular focus on innovation in imaging technologies and biomarker discovery. We examine the epidemiologic and mechanistic aspects of early disease, review the latest radiologic techniques and molecular candidates, and identify knowledge gaps and future research priorities. In addition, we discuss therapeutic implications, particularly the role of early lifestyle interventions, enzyme supplementation, and future antifibrotic or immunomodulatory agents.

## 2. Early Chronic Pancreatitis: Definition and Diagnostic Criteria

**Figure 2:**
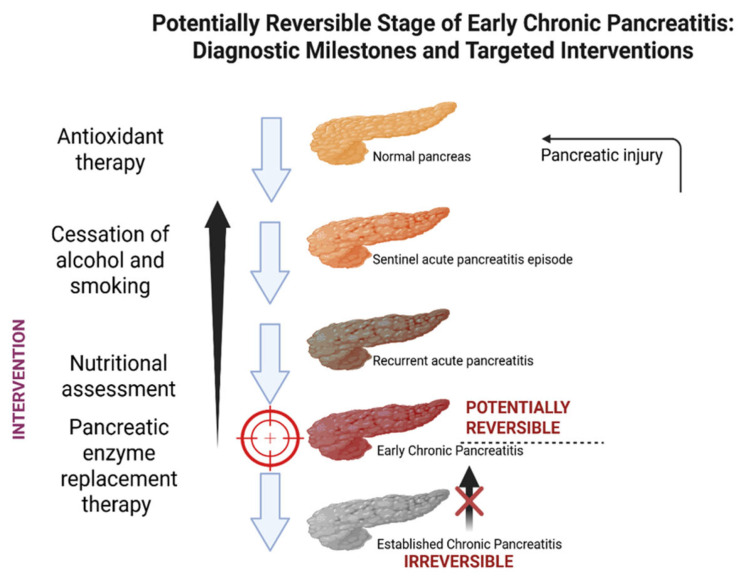
The progression from a normal pancreas to established CP typically occurs through a series of clinical and structural transitions, beginning with a sentinel acute pancreatitis episode, followed by recurrent acute pancreatitis, then ECP, and ultimately leading to irreversible CP.

The concept of ECP has emerged from the growing realization that CP is not a static endpoint but a progressive inflammatory condition that begins before irreversible morphological damage is apparent [12]. The official recognition of ECP as a clinical entity was formally introduced by the JPS in 2019. Since then, we refer to ECP as a clinically and biologically distinct phase in the progression from RAP to overt CP. It is characterized by subtle morphological, functional, or biochemical pancreatic changes that precede the irreversible fibrosis and insufficiency seen in late-stage CP [13]. At this stage, it is important to distinguish between the conceptual background—defining ECP as an early, potentially reversible stage—and the available evidence from cohort studies or diagnostic trials. The following section therefore synthesizes published results separately, focusing on imaging, biomarkers, and longitudinal outcomes. Identification of ECP may allow for timely intervention to slow disease progression (Figure 2).

ECP is estimated to affect 10–20% of patients initially diagnosed with RAP, especially in alcohol-related cases [24]. There is a risk-based approach, as suggested by the International Consensus Guidelines. The consensus introduced the term “early CP” as a condition where genetic, environmental, and inflammatory factors are present, but irreversible fibrosis has not yet occurred [12]. Unlike the JPS imaging-based system, this model proposed a multifactorial, progressive definition of CP—supporting the idea that ECP exists on a continuum of pancreatic disease, even when classical imaging is not yet definitive [13]. The main conceptual elements of this consensus are summarized in Table 1.

Currently, the diagnostic criteria utilized for ECP are those employed by JPS and rely on a combination of clinical (recurrent epigastric or back pain, elevated pancreatic enzyme levels in serum or urine, evidence of pancreatic exocrine insufficiency, continuous heavy alcohol consumption ≥ 60 g/day, or genetic predisposition and history of acute pancreatitis) and radiological features (irregular dilatation of ≥3 side branches of the main pancreatic duct), preferably assessed by MRI/MRCP, EUS, or ERCP. A positive diagnosis requires the presence of radiological changes and at least two additional clinical features [25,26].

Furthermore, a review of the most relevant studies on ECP published between 2009 and 2025 was performed, focusing on progression from RAP, diagnostic imaging findings, biomarker developments, and long-term outcomes. The selected literature is synthesized in Table 2, providing a comparative and up-to-date evidence base.

From a conceptual perspective, ECP has traditionally been regarded as a precursor to irreversible pancreatic damage [32,33,34,35,36].

However, recent prospective studies and cohort analyses demonstrate that its trajectory is heterogeneous, with a proportion of patients experiencing regression or stabilization under lifestyle modification and structured surveillance [37].

Sheel et al. [38] found that in patients with minimal change EUS findings, 30% progressed to definite CP. The main incriminated factors were alcohol and tobacco use. Beyer et al. [39] emphasized interdisciplinary care and structured follow-up as critical in preventing irreversible pancreatic damage. Annual assessments for pain, malnutrition, exocrine/endocrine dysfunction, and lifestyle risk factors are essential. Schneider et al. [40] followed a cohort of 350 patients with MRI-defined ECP over 5 years and found that only 7% progressed to definite CP, while nearly 40% demonstrated regression on follow-up imaging, particularly those with sustained abstinence from alcohol and tobacco. Similar results were reported by Lee et al. [41] in a prospective observational study of 220 patients with EUS-detected early pancreatic irregularities. Among them, 28% progressed to CP within 3 years; cessation of alcohol use was associated with a 2.5-fold lower progression rate. De Rijk et al. [42] showed that smoking was an independent predictor of structural progression. Evidence from animal models supports the hypothesis that repeated acute injury may drive the transition to early fibrosis and chronic disease. In experimental models, recurrent acute pancreatitis episodes induce progressive periductal and parenchymal fibrosis, inflammatory cell infiltration, and acinar atrophy, mimicking early human disease [31]. Additional preclinical studies demonstrated that intra-acinar activation of proteases and sustained inflammatory signalling are key drivers of this process [43].

Ge et al. [44] reported that regular annual surveillance with combined high-resolution MRCP and EUS improves early detection of subtle ductal changes and may identify regression before fibrosis is irreversible.

The evolution of ECP is not unidirectional. A substantial subset of patients may avoid progression to established chronic disease, particularly through alcohol and smoking cessation, early detection via imaging, and multidisciplinary follow-up. These findings emphasize the need to redefine ECP not merely as a transitional state but as a potentially reversible condition when managed proactively.

## 3. Diagnostic Modalities

### 3.1. Imaging Techniques

The imaging diagnosis of ECP is challenging, situated at the borderline between acute inflammatory episodes and established pancreatic fibrosis. Unlike AP, where contrast-enhanced computed tomography (CECT) is used to evaluate severity and necrosis, or advanced CP, where calcifications, atrophy, and pseudocysts are readily visualized, ECP requires imaging tools sensitive enough to detect subtle, early-stage changes before irreversible damage occurs [45,46,47,48].

EUS is currently considered the most accurate imaging modality for ECP, offering sensitivity between 81–84% and 90–100% specificity, as shown in recent studies [35,48]. MRI achieves 77–78% sensitivity and 83–96% specificity, while transabdominal ultrasound has lower sensitivity (67–69%) but high specificity (90–98%). CT shows limited sensitivity in ECP (20–70%) but excellent specificity (91.9–100%). EUS enables high-resolution visualization of minor ductal and parenchymal alterations, such as hyperechoic foci, lobularity, and early ductal irregularities [49]. However, traditional EUS evaluation can be subjective and interobserver-dependent [50,51]. Nagahama et al. [41] studied asymptomatic patients with pancreas divisum and found that 47% had EUS features consistent with ECP, primarily hyperechoic foci (82%) [52].

Histological examination remains the gold standard for confirming ECP, although it is rarely performed due to its invasive nature. Early lesions are characterized by periductal and interlobular fibrosis, acinar cell atrophy, inflammatory cell infiltration (particularly lymphocytes and macrophages), and distortion of small intralobular ducts. When EUS findings are inconclusive, particularly in the differentiation of early chronic pancreatitis from other focal pancreatic masses, tissue sampling can provide critical diagnostic information. In this context, fine-needle aspiration (FNA) and fine-needle biopsy (FNB) are valuable adjuncts to imaging [53]. A recent network meta-analysis including 27 studies and 4857 patients demonstrated that EUS-FNB yields significantly higher histologic adequacy (risk ratio ~1.25) and overall diagnostic accuracy (87–92% vs. 80–85% for FNA), while requiring fewer needle passes to establish a diagnosis. Complication rates were low and comparable between the two techniques. These findings support the preferential use of FNB when tissue acquisition is warranted for differential diagnosis, especially in cases where malignancy cannot be excluded. Thus, while EUS remains central for early detection, selective tissue sampling serves as an important complementary tool to overcome its limitations in distinguishing ECP from pancreatic neoplasia [53]. Progressive replacement of acinar tissue by fibrous tissue leads to irreversible architectural changes [54]. Recent studies underline the importance of correlating histological findings with clinical and imaging criteria to improve diagnostic accuracy and to better define early stages of the disease [54].

**Figure 3:**
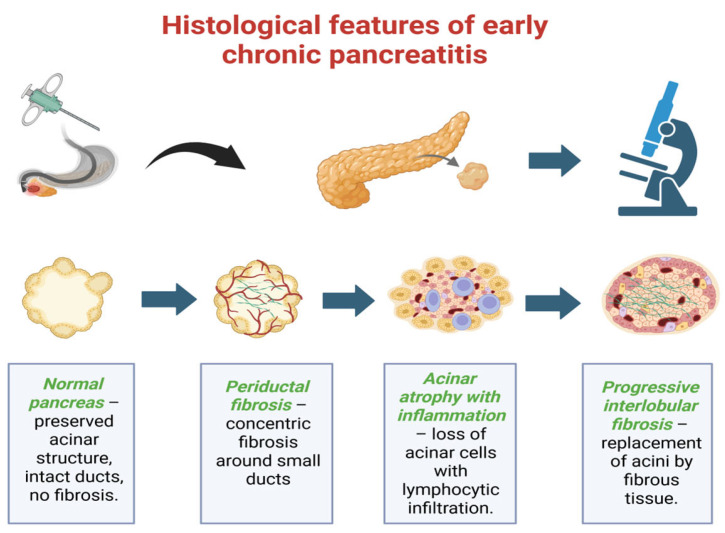
Histological features of early chronic pancreatitis.

When EUS findings are inconclusive or when differentiation from focal pancreatic masses is necessary, tissue sampling by fine-needle aspiration (FNA) or fine-needle biopsy (FNB) may provide additional diagnostic yield. A recent network meta-analysis confirmed that EUS-FNB achieves superior histologic adequacy and diagnostic accuracy compared with FNA for solid pancreatic lesions [54]. In the context of suspected early chronic pancreatitis, tissue sampling should be reserved for selected cases where malignancy cannot be excluded, serving as a valuable complement to imaging [55]. As shown in Figure 3, the progression begins with preserved acinar and ductal structures, followed by the development of periductal fibrosis, acinar atrophy with inflammatory infiltration, and finally interlobular fibrosis with distortion of the normal architecture. This figure summarizes the stepwise microscopic changes characteristic of the early stages of the disease.

To overcome these limitations, elastography-based EUS methods have been developed, which measure tissue stiffness and provide a functional assessment of fibrotic remodelling. Real-time elastography has shown improved sensitivity (up to 95%) but moderate specificity (~53%) [56]. These findings suggest elastography is particularly useful in early-stage disease, where standard echotexture changes are minimal. Contrast-enhanced EUS (CE-EUS) further improves diagnostic confidence by assessing perfusion abnormalities, especially when differentiating inflammatory versus fibrotic changes [57].

MRI and magnetic resonance cholangio-pancreatography (MRCP), especially with high-resolution T2-weighted sequences, represent important non-invasive tools for ductal assessment. MRI achieves 77–78% sensitivity and up to 96% specificity in detecting early parenchymal changes [16,58]. Furthermore, T1-weighted signal intensity ratio (SIR) between the pancreas and spleen has been correlated with exocrine dysfunction, with SIR < 1.2 associated with significant secretory insufficiency [59]. Such findings suggest MRI can indirectly quantify functional compromise in ECP.

Shear-wave elastography (SWE), a transabdominal ultrasound-based technique, has also gained traction as a less invasive and repeatable modality. Studies have shown SWE achieves 76% sensitivity and 88% specificity, providing an option for surveillance in resource-limited or non-invasive settings [60]. High-resolution secretin-stimulated MRCP can reveal subtle ductal changes and has shown promise in tracking disease regression in patients under risk-reduction strategies [61].

Recent studies emphasize the utility of longitudinal imaging follow-up, especially using combine EUS and MRI, to document regression or progression. Annual surveillance imaging has been shown to identify ductal changes before clinical deterioration, offering opportunities for early intervention [61]. Notably, AI-enhanced image interpretation is emerging as a tool for improving diagnostic reproducibility. In a multicentre validation study, AI-based EUS interpretation reduced interobserver variability and improved diagnostic accuracy in early fibrosis detection [62]. Despite these advances, imaging research in ECP is still affected by major limitations. Protocols differ considerably between studies, cut-off values for elastography or MRI parameters are not standardized, and inter-observer variability remains significant. These methodological inconsistencies limit external comparability and delay the establishment of universally accepted diagnostic thresholds.

The Rosemont criteria remain widely used for the EUS-based diagnosis of chronic pancreatitis. However, their sensitivity and specificity in early disease are variable. A large multicenter analysis reported sensitivities between 61 and 84% and specificities of 72–100%, depending on the diagnostic threshold applied [38]. In ECP, these criteria may overestimate subtle parenchymal abnormalities, while inter-observer variability remains significant [63]. Recent reviews have further emphasized that Rosemont scoring, while systematic, is prone to both inter-reader subjectivity and limited reproducibility, and thus should not be used in isolation for early-stage disease [63,64]. Complementary imaging modalities such as MRI and elastography are increasingly advocated to improve diagnostic confidence and refine disease stratification [63]. Moreover, Ishikawa et al. highlighted the value of combining EUS with MRI and emerging imaging biomarkers to enhance diagnostic precision in ECP [63]. Overall, although Rosemont scoring provides a structured framework, its diagnostic accuracy in early stages is still limited, and integration with multimodality imaging is recommended [63,64]. ECP imaging serves not only as a diagnostic modality, but also as a longitudinal management tool within precision medicine frameworks [65]. A comprehensive summary of imaging modalities used in the diagnosis of ECP was provided by Ge Q.C. et al. [63], who emphasized the complementary role of transabdominal ultrasound, MRI/MRCP, and EUS—including elastography and contrast-enhanced techniques—in detecting subtle structural changes before irreversible fibrosis develops.

The review assessed the diagnostic capabilities, strengths, and limitations of each modality in ECP, serving as a valuable reference in clinical decision-making. The key features and comparative performance of these imaging techniques are detailed in Table 3.

### 3.2. Biomarkers

In certain chronic fibrosing diseases, such as liver or pulmonary fibrosis, serum biomarkers have proven useful for detecting early-stage progression before irreversible damage occurs. In the context of pancreatitis, reliable and validated biomarkers could provide clinicians with tools to detect subclinical inflammation, early fibrosis, and guide monitoring or treatment response [64].

C-reactive protein (CRP) is a recognized marker of systemic inflammation in AP, with levels ≥ 190 mg/L at 48 h predicting severe outcomes with high sensitivity [58]. However, its utility in early fibrotic detection remains limited [66,67].

Emerging evidence suggests that inflammatory cytokines such as interleukin-6 (IL-6) and tumour necrosis factor alpha (TNF-α) may provide discriminatory power between RAP and ECP. In a recent review by Poulsen et al. [22], authors identified the elevation of IL-6 and sCD163 levels (macrophage activation markers) correlated with immune-driven changes in ECP. However, variability across patient populations and technical challenges in standardizing cut-offs pose significant challenges [42].

Further research has expanded into matrix MMPs, especially MMP-9 and their inhibitors such as TIMP-1, which are involved in pancreatic remodelling and fibrosis. Elevated MMP-9/TIMP-1 ratios have been shown to precede imaging-detectable changes in pancreatic structure, suggesting a potential role in early detection [68,69]. The PROCEED study utilized machine learning-based immunoassay profiling and identified IL-6, MCP-1, IL-8, and VEGF as part of a reproducible inflammatory signature across the AP-RAP-CP spectrum. These markers, particularly when used in combination, may help predict progression and distinguish subclinical fibrosis from benign recurrence [70]. Oxidative stress markers like TGF-β1, malondialdehyde (MDA), and 8-isoprostane have also been reported in small cohorts to correlate with disease severity and progression. Moreover, adipokines such as leptin and adiponectin—linked to metabolic dysfunction—have demonstrated elevated levels in CP and RAP patients, reflecting systemic metabolic inflammation [23].

Other studies also explored non-coding RNAs, especially miRNAs. For example, elevated miR-126-5p and miR-130b-5p have been detected in early stages and correlate with inflammatory burden [71,72].

Notably, phospholipase D2 was recently reported to decrease significantly in patients with CP and correlate with reduced neutrophil migration, offering a mechanistic link to tissue injury and inflammation in the pancreas [73]. Similarly, sCD14 and CD206—macrophage-related markers—have been associated with immune phenotypes in patients with evolving CP [74].

Despite these advances, no biomarker or panel is yet validated for routine clinical use in ECP [75]. However, evidence increasingly supports the concept that combined biomarker profiling may assist in identifying patients at high risk of progression, especially when imaging findings are equivocal. Serial biomarker monitoring could also serve as a dynamic tool to assess disease trajectory, particularly in research settings [76].

Nevertheless, current biomarker research remains preliminary. Most studies are exploratory, include small and heterogeneous cohorts, and often apply non-standardized assays with variable cut-off thresholds. This methodological variability limits reproducibility and hinders comparison across studies. Furthermore, the majority of data are cross-sectional, lacking longitudinal validation to establish predictive value. As a result, no biomarker—whether cytokine-based, matrix-derived, or microRNA—can yet be considered ready for clinical translation in early chronic pancreatitis.

Additional work in AP cohorts indicates that miRNAs like miR-19a, miR-143, and miR-374-5p correlate with disease severity and outperform traditional CRP or scoring systems in early detection of severe acute episodes [77]. MicroRNAs (miRNAs) are emerging as promising non-invasive biomarkers in pancreatitis research, capable of reflecting early disease mechanisms at the molecular level. Several recent studies have identified specific miRNA signatures—such as hsa-miR-221, miR-130a, miR-199a-3p, and miR-1471—that differentiate early CP from healthy controls with high accuracy, even before morphological changes manifest on imaging [78,79]. Hegyi et al. [31] conducted a translational mouse-human model demonstrating that ≥3 episodes of acute pancreatitis can induce early CP-like ductal changes even without overt imaging findings. This insight supports the rationale that miRNA dysregulation may precede or accompany structural remodelling in early disease stages.

Furthermore, exosome-derived miRNA panels are being explored for detecting subclinical pancreatic injury, drawing on technology proven in early pancreatic cancer detection [32,80,81,82]. Changes in miRNA occur alongside protease activation, inflammatory cytokine shifts, and ductal stress pathways—categories central to disease initiation according to mechanistic definitions [83].

Overall, although still investigational, miRNAs hold substantial promise for early detection of ECP, serving as minimally invasive markers of ductal injury, inflammation, and fibrosis before irreversible changes occur [84].

## 4. Therapeutic Strategies in Early Chronic Pancreatitis

### 4.1. Lifestyle Interventions

Lifestyle interventions remain the cornerstone of therapeutic management in ECP, targeting modifiable risk factors such as alcohol and tobacco use. Conceptually, early risk reduction is expected to slow progression [85]. Supporting this, multiple high-quality prospective studies and controlled interventions have demonstrated that abstinence from alcohol following an acute or recurrent episode significantly reduces recurrence rates. Up to 79% of patients reported maintained abstinence at one month, accompanied by significant reductions in biomarkers such as GGT levels and mean corpuscular volume, indicating early behavioural and biochemical response [86]. Although long-term data remain limited, cohort analyses show that structured cessation support reduces recurrence of alcohol-related pancreatitis by over 50%, with recurrence rates dropping from ~30% to below 10% over two years of follow-up [87].

Smoking cessation is also a crucial determinant of disease trajectory. A dose–response relationship between pack-years and AP severity has been observed, with odds ratios increasing from 3.8 for light smokers to over 8 for heavy smokers [88,89,90]. In CP cohorts, smokers consistently present more severe pain levels, more frequent exacerbations, and poorer quality of life, highlighting the need for early tobacco abstinence [87,91].

Overall, combined cessation of alcohol and tobacco is the most effective non-pharmacological intervention in ECP. Data consistently show that patients who remain abstinent have lower imaging progression, reduced biomarker activity, and improved functional status. This underlines the need to embed lifestyle counselling into standard follow-up protocols [92,93,94,95].

### 4.2. Nutritional Optimization and Metabolic Correction

Several recent cohort studies show that targeted dietary regulation—and correction of metabolic derangements—can improve exocrine function, reduce relapse frequency in RAP, and slow structural progression.

A multicentre prospective analysis found that low-fat diets enriched with medium-chain triglycerides (MCTs) led to improved pain control and reduced steatorrhea in 68% of ECP patients after six months [95]. Another study demonstrated that early initiation of pancreatic enzyme replacement therapy (PERT) enabled a 45% improvement in fat absorption as measured by coefficient of fat absorption (CFA), delaying nutritional decline [96].

Obesity and insulin resistance are increasingly recognized contributors to ECP progression [96]. Mendelian randomization and cohort data both confirm that correcting metabolic syndrome features—especially waist-adjusted BMI and visceral adiposity—alleviates inflammatory cytokine activation and lowers progression risk [96]. In a randomized controlled trial, patients with RAP who achieved >5% body weight reduction via a combined dietary and GLP-1 agonist regimen had an 18% lower recurrence at one year than controls [96].

A recent observational study among ECP patients with malnutrition reported that early supplemental pancreatic enzymes, coupled with oral nutritional supplementation (ONS) rich in antioxidants, significantly improved nutritional scores (SGA) and decreased serum oxidative stress markers.

Furthermore, micronutrient deficiencies—particularly vitamins D and E, selenium, and zinc—are common in ECP and have been associated with impaired immune response and slower regeneration. Supplementation of vitamin D to achieve serum levels > 50 ng/mL reduced inflammatory markers (CRP, IL-6) in a cohort of Indian ECP patients [97].

In children with genetic predisposition to ECP—such as PRSS1 or SPINK1 carriers—growth impairment and endocrine dysfunction are mitigated by early nutritional intervention, including PERT and dietician-led support [98]. A randomized controlled trial assessing Mediterranean-style dietary counselling in RAP/ECP patients showed a notable decrease in oxidative stress markers and inflammatory cytokines over six months [98].

Strategies to control hyperlipidemia—particularly high triglyceride levels—through omega-3 supplementation and statins resulted in improved disease stability and lower relapse in ECP patients with metabolic-associated pancreatitis [99]. A meta-analysis combining dietary interventions and lipid-lowering therapy showed a 31% decrease in structural progression on imaging over two years in early-stage patients [100].

Collectively, these findings underscore that integrating nutritional rehabilitation, enzyme support, and metabolic regulation into standard care for ECP can alleviate symptoms, support recovery, and delay—or potentially prevent—progression to overt CP.

### 4.3. Enzyme Supplementation: Timing and Rationale

PERT plays an important dual role in ECP. It improves digestion and may reduce progression of pancreatic injury by reducing autodigestion and inflammation. Recent studies have emphasized the benefits of PERT, even in patients with ascertained malabsorption [101].

A study showed that starting PERT within three months of an initial AP episode improved fat absorption by 45% and significantly reduced abdominal pain and anthropometric decline after six months [102]. ECP patients treated with high-dose PERT had higher levels of fecal elastase and lower inflammatory markers compared to those in placebo group [102].

PERT initiation (within 30 days of acute episode) was associated with a 35% reduction in recurrent episodes over two years and fewer ECP-related hospitalizations [103]. Combination therapy with proton pump inhibitors improves enzyme delivery and symptom control, particularly in patients with reflux or gastritis [104].

PERT combined with dietary modification reduced both pain scores and inflammatory biomarker levels (IL-6, CRP) more than dietary change alone, suggesting potential anti-inflammatory benefits [105]. PERT initiation in patients with imaging or functional signs of exocrine insufficiency may bring benefits in order to preserve nutritional status and it may slow structural progression [106]. Enzyme doses adjusted to body weight (500 units lipase/kg per meal) correlated with better control of malabsorption and symptom relief. A specialized formulation using enteric-coated microspheres improved patient adherence and gastrointestinal tolerability, reducing the incidence of abdominal discomfort and nausea [107].

PERT initiation within six months of RAP/ECP onset was associated with fewer relapses and better nutritional parameters (such as albumin or vitamins D/E) with no increased adverse events [99,103,108]. These findings support treatment by PERT in ECP can be useful in maintaining nutrient absorption and potentially to reduce inflammatory progression [109].

### 4.4. Pain Control: Step-Up Approach

Pain in ECP is usually reported as intermittent, epigastric, and related to food ingestion. It may significantly impair quality of life and may precede physical decline [110]. A step-up pain management strategy begins with lifestyle modification and dietary changes, then progresses to pharmacologic and, if needed, endoscopic or surgical management.

As a first line approach, pharmacotherapy includes non-opioid analgesics and PERT, which decrease pain by reducing enzyme release. If pain is persistent, low-dose neuropathic agents such as gabapentin can be prescribed. A study showed that 68% of ECP patients achieved ≥30% pain reduction with combined enzyme and gabapentin therapy within six months, without opioid escalation [111]. If necessary, celiac plexus block (CPB) or thoracoscopic splanchnicectomy, particularly can be used for patients with refractory pain and minimal ductal abnormalities. In a multicentric randomized study, CPB provided up to 6 months of pain relief in 55% of patients, delaying need for opioids [112]. Similarly, endoscopic ultrasound–guided CPB showed efficacy and good tolerability in early disease phenotypes [113]. Systematic reviews now support stepwise escalation, with opioids reserved for refractory cases after elimination of metabolic, anatomical, and functional contributing factors [114]. Behavioural therapies, including cognitive behavioural therapy and biofeedback, also demonstrated benefit, improving pain and reducing perceived severity [115].

Pain control strategies should be individualized: neuropathy modulators in neuropathic pain, PERT in enzymatic pain patterns, and procedural interventions (e.g., CPB) when pain persists despite medical management [116]. The initiating of neuropathic agents within three months of symptomatic onset reduced opioid dependency by 40% at one year [117].

New therapies such as intrathecal targeted drug delivery and neurolytic nerve ablation are under investigation for severely affected cases, though data are currently limited [118]. Multimodal pain control emphasizing non-opioid pharmacotherapy, targeted procedures, and psychosocial support [118].

### 4.5. Antifibrotics and Immune Modulators

Fibrosis is the defining feature of irreversible pancreatic damage. In ECP, strategies targeting fibrogenesis and immune dysregulation represent a potential avenue to delay or halt progression. Several recent preclinical and translational studies have focused on disrupting the pancreatic fibrotic cascade at various levels.

Small-molecule inhibitors of TGF-β receptors have demonstrated efficacy in preclinical ECP models by halting collagen deposition and preserving ductal integrity [119]. MMP-9 inhibitors have reduced serum MMP-9/TIMP-1 ratios and improved symptom scores in early human studies [120]. Anti-IL-6 monoclonal antibodies such as tocilizumab have shown reductions in inflammatory cytokines and stabilization of fibrosis markers on MR elastography in small open-label trials [120]. Mesenchymal stem cell (MSC) therapy has demonstrated immune-modulatory effects by reducing TNF-α, IL-6, and TGF-β1 in early-stage fibrosis when infused intra-arterially [121].

Additionally, angiotensin pathway inhibitors such as losartan have been associated with decreased pancreatic stiffness and stellate cell activation in patients with early-stage CP [45]. New-generation antagomirs, particularly those targeting miR-21 and miR-199a, have successfully reversed fibrosis in murine models through suppression of proteotoxic and fibrogenic pathways [122]. Moreover, targeted suppression of the NF-κB signalling pathway has demonstrated attenuation of inflammatory activation and reduced parenchymal damage in RAP-to-CP progression models [123,124].

Inhibition of the JAK-STAT axis has also emerged as a novel therapeutic direction, with JAK1/2 inhibitors leading to reduced expression of α-SMA and collagen in experimental pancreatitis [121]. Unexpectedly, SGLT2 inhibitors—commonly used in diabetes—have been shown to exert antifibrotic effects by reducing pancreatic inflammation, likely due to decreased oxidative stress and improved metabolic parameters [125]. Furthermore, pirfenidone analogues, already approved for pulmonary fibrosis, have shown promising early results in CP trials, with observed reductions in fibrotic biomarkers and improvement in exocrine function [123]. Lastly, experimental blockade of CTGF (connective tissue growth factor)—a master regulator of fibrogenesis—has delayed progression in preclinical models of pancreatic injury [124].

Although no antifibrotic or immune-modulating agent is currently approved for ECP, these emerging studies open possibilities for precision-guided interventions, especially in patients identified as high risk for progression through genetic, imaging, or biomarker profiles.

### 4.6. Role of Endoscopy

Endoscopic therapy plays a central role in the management of pain and complications in chronic pancreatitis, particularly in patients with obstructive disease. Advances in stent technology have improved outcomes: fully covered self-expandable metal stents (FCSEMS), specifically designed for pancreatic duct strictures, have demonstrated high technical success and durable pain relief in multicenter prospective studies, while also reducing the need for repeated procedures [126]. Plastic stents remain widely used, but they are associated with shorter patency and higher recurrence rates compared with newer FCSEMS. Such innovations suggest a gradual shift toward metal stents in selected patients, although long-term data are still limited.

**Figure 4:**
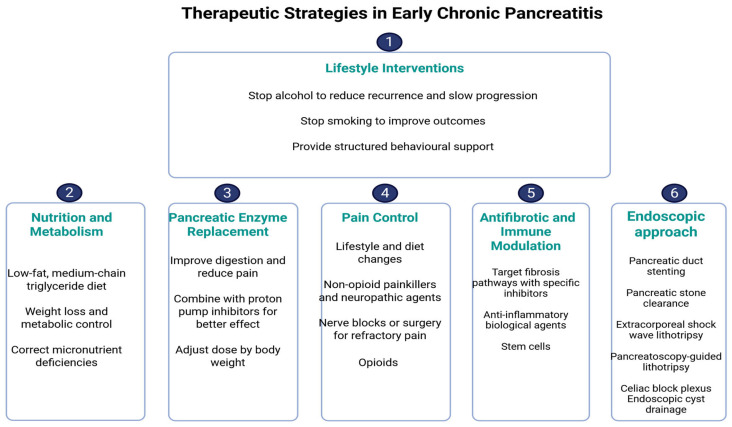
Therapeutic strategies in ECP.

For pancreatic duct stones, extracorporeal shock wave lithotripsy (ESWL) combined with endoscopic clearance continues to represent the standard approach. However, digital single-operator pancreatoscopy with intraductal lithotripsy has emerged as a valuable tool in refractory cases, achieving higher ductal clearance rates and symptom improvement compared with conventional methods [122]. Updated guidelines from the American Gastroenterological Association (AGA) and the American Society for Gastrointestinal Endoscopy (ASGE) recommend an “endoscopy-first” approach for suitable candidates, reserving surgery for patients who fail to achieve durable relief [127]. Nevertheless, long-term follow-up of randomized clinical trials, such as the ESCAPE trial, indicates that while endoscopy can achieve meaningful pain relief and quality-of-life benefits, repeated interventions are often required, and early surgery may offer superior durability in selected patients [128]. Recent studies emphasize that therapeutic endoscopy in chronic pancreatitis is moving toward more personalized strategies, combining advanced stent technology, intraductal lithotripsy, and minimally invasive approaches, with the goal of optimizing pain control while delaying or avoiding surgical intervention [129]. Figure 4 summarizes the proposed therapeutic approach in ECP.

## 5. Limitations

This review has several limitations. Evidence on early chronic pancreatitis remains heterogeneous and largely observational. Imaging studies differ in protocols, scoring systems, and thresholds, reducing reproducibility across centres. Biomarker research is especially limited: most studies are exploratory, based on small cohorts, with variable assays and inconsistent cut-off values.

Many candidates (e.g., IL-6, sCD163, MMP/TIMP ratios, microRNAs) lack prospective validation and standardization, which prevents translation into clinical use. Differences in sampling methods, timing of collection, and population characteristics further reduce comparability. Genetic and molecular studies are often restricted to selected groups and may not reflect the general population. Finally, as a narrative review, selection bias cannot be fully excluded. These limitations highlight the need for multicenter studies, harmonized biomarker assays, and standardized diagnostic frameworks.

## 6. Future Perspectives and Research Gaps

Despite significant progress in understanding ECP, several gaps remain. Future research must address these systematically to enable reliable diagnosis, risk stratification, and timely therapeutic interventions. Four main domains require focused investigation: biomarkers, imaging, precision medicine with registries, and therapeutic innovation.

### 6.1. Biomarker Validation

One of the major unmet needs in ECP is the absence of clinically validated biomarkers. Current evidence is largely limited to exploratory studies reporting associations between inflammatory cytokines (IL-6, sCD163), fibrotic mediators (MMP-9, TIMP-1, TGF-β1), and microRNAs with early structural changes [130]. However, these findings come from heterogeneous populations, employ non-standardized assays, and frequently use variable cut-off values, which limits reproducibility. Furthermore, most studies are cross-sectional, providing only a static view of disease biology. Future work should move toward large, multicenter, prospective cohorts where biomarkers are collected longitudinally and correlated with imaging and functional outcomes. Such studies should also harmonize pre-analytical and analytical methods to ensure comparability. The integration of multi-marker panels, possibly including circulating proteins, microRNAs, and exosomal signatures, may yield higher accuracy than isolated markers, but requires robust external validation before clinical use [131].

### 6.2. Imaging Standardization

Although EUS, MRI-based techniques (T1 mapping, MR elastography), and elastography have substantially improved sensitivity for early parenchymal and ductal changes, they are still hampered by significant inter-observer variability and a lack of standardized scoring criteria. Quantitative cut-offs differ across centres, and imaging protocols are rarely harmonized. Future research should therefore focus on developing consensus-based imaging protocols supported by multicenter validation. Particular attention should be given to standardizing EUS elastography, which has shown promise in detecting early fibrosis, but currently lacks uniform technical and interpretive guidelines [132]. Artificial intelligence-based interpretation could also reduce operator dependence, but requires validation across diverse datasets [133].

### 6.3. Precision Medicine and Registries

The natural history of ECP is heterogeneous: while some patients progress to established CP, others experience stabilization or even regression under favourable lifestyle and clinical conditions. This variability highlights the need for precision medicine approaches that integrate genetic predisposition (e.g., PRSS1, SPINK1, CTRC variants), biomarker profiles, and imaging phenotypes into predictive models [134]. Such models could enable individualized risk stratification and targeted early intervention. These approaches require data from large longitudinal registries [135]. 

### 6.4. Therapeutic Innovation

Currently, lifestyle modification (alcohol and smoking cessation) remains the cornerstone of management in ECP. Yet there is increasing interest in antifibrotic and immune-modulating strategies aimed at halting or even reversing pancreatic fibrosis. Preclinical data suggest that targeting pathways such as TGF-β, NF-κB, and JAK/STAT may attenuate fibrogenesis, while mesenchymal stem cell therapy and microRNA-directed interventions show promise in experimental models [136,137]. Translating these strategies into clinical practice will require carefully designed early-phase trials conducted in well-defined ECP cohorts. These trials must incorporate validated biomarkers and imaging endpoints to identify responsive subgroups and avoid overtreatment. Importantly, recent translational studies suggest that fibrosis reversal may still be achievable in early disease, a finding that reinforces the importance of timely detection and intervention [138].

## 7. Conclusions

This review provides a comprehensive and up-to-date synthesis of recent advances in the diagnosis and management of ECP. By integrating data from imaging, biomarkers, and genetic findings, it offers a multidisciplinary perspective that supports early risk stratification. The inclusion of both established and emerging diagnostic tools, sauch as elastography, MRI-T1 mapping, and microRNAs, enhances its translational value. This review also highlights the clinical relevance of lifestyle interventions and anticipates future developments involving artificial intelligence and precision medicine.

Significant heterogeneity across studies still persists regarding diagnostic criteria, and the lack of large-scale prospective validation for many proposed biomarkers poses an important drawback. The predominance of observational and cross-sectional studies limits causal inferences. Moreover, inter-observer variability in EUS and the absence of standardized imaging protocols may affect reproducibility. Further longitudinal research is needed to establish validated thresholds for early intervention and to unify diagnostic frameworks across clinical settings.

## Figures and Tables

**Table 1:** Diagnostic criteria for ECP proposed by the International Consensus Guidelines.

Domain	Consensus Statement/Conclusion	Clinical Implication
Definition	ECP is a stage of disease characterized by ongoing pathogenic mechanisms without end-stage damage.	Emphasizes a dynamic process; shifts focus from imaging to mechanism-based definition.
Pathophysiology	Chronic pancreatitis arises from repeated injury, inadequate repair, and progressive fibrosis.	Highlights the need for early identification of risk factors and repeated inflammatory events.
Diagnostic Focus	Traditional imaging findings are not required to define ECP.	Diagnosis should be based on mechanistic understanding, not just structural changes.
Mechanistic Model	CP should be defined by underlying biological processes (e.g., inflammation, fibrosis, genetics).	Supports use of biomarkers and risk stratification tools to guide diagnosis and therapy.
Role of Genetics	Genetic variants (e.g., PRSS1, SPINK1, CFTR) modify susceptibility and progression risk.	Genetic testing is useful in early or idiopathic cases, especially in young patients.
EUS and Imaging	Imaging may appear normal or nonspecific in ECP.	Reliance solely on CT/MRI may delay diagnosis; EUS and secretin-MRCP offer more sensitivity.
Biomarkers	Need for validated biomarkers of early-stage disease and progression.	Research should prioritize non-invasive diagnostic and prognostic molecular tools.
Clinical Presentation	Symptoms in early CP may mimic functional GI disorders.	Requires low threshold of clinical suspicion; overlap with irritable bowel syndrome/dyspepsia is frequent.
Goal of Diagnosis	To identify at-risk individuals before irreversible injury occurs.	Enables potential disease modification through early lifestyle and therapeutic intervention.
Prognostic Stratification	Disease evolution is heterogeneous; not all patients with RAP will progress to CP.	Risk prediction tools should integrate clinical, genetic, and biomarker data.

**Table 2:** Review of current research studies regarding ECP.

No.	First Author (Year)	Country	Study Type	No. Patients/Data	Main Objective/Imaging	Key Findings
1	Japanese Pancreas Society (2009) [27]	Japan	Expert consensus	N/A	EUS	Proposed first diagnostic criteria for ECP based on EUS and clinical features.
2	Whitcomb et al. (2018) [12]	International	Expert consensus	N/A	Multimodal	Defined the concept of early CP and proposed mechanistic classification and diagnostic model.
3	Stevens T. et al. (2009) [28]	USA	Narrative review	N/A	EUS	Highlighted EUS as sensitive but operator-dependent; advocated for integrated imaging criteria.
4	Tirkes et al. (2019) [29]	USA (Indiana University)	Prospective	69	MRI T1 mapping	T1 mapping identified significant differences between controls, ECP, and definite CP.
5	Masamune et al. (2019) [30]	Japan	Multicentre cohort study	83	MRI + EUS	Found that 4.8% progressed to CP while 36.1% regressed; lifestyle changes were protective.
7	Hegyi et al. (2021) [31]	Hungary	Cross-species study	Human + mouse model	Histology + Imaging	≥3 AP episodes were associated with irreversible fibrotic changes in pancreas tissue.
8	Yamamiya et al. (2022) [3]	Japan	Retrospective cohort	100	Clinical criteria + EUS	High AUDIT-C scores predicted CP progression in alcohol-related ECP.
9	Poulsen et al. (2024) [22]	Romania	Narrative review	N/A	Biomarkers	Reviewed IL-6, sCD163, MMP-9 as potential markers of ECP progression.

**Table 3:** Imaging techniques for ECP detection.

Modality	Purpose/Description	Advantages	Limitations	Clinical Utility
Transabdominal Ultrasound (US)	Initial assessment tool for pancreas (parenchyma and duct).	Non-invasive, inexpensive, widely available.	Limited by bowel gas; sensitivity reduced in early/mild disease.	First-line screen; follow-up with advanced imaging if suspicion remains high.
CE-US	Assesses pancreatic perfusion and microvascular changes.	Can detect inflammation, vascular alterations.	Operator- and equipment-dependent; less standardized for CP.	Adjunctive tool in experienced centres, notably for vascular findings.
EUS	High-resolution imaging of pancreatic parenchyma and ducts.	Excellent spatial resolution; detects subtle parenchymal/duct abnormalities.	Interobserver variability; invasive, operator-dependent; sedation required.	Gold standard for early structural detection; ideal for borderline cases.
EUS Elastography	Measures tissue stiffness to detect early fibrosis.	Quantitative assessment; identifies preclinical fibrotic changes.	Lacks standardized cut-offs; technique-dependent; evolving evidence.	Promising tool for fibrosis staging alongside conventional EUS.
EUS-guided nCLE	In vivo microscopic imaging via confocal laser endomicroscopy.	Cellular-level resolution; distinguishes fibrosis from other lesions.	Invasive (requires FNA), risk of bleeding/infection, expensive.	May aid differentiation from malignancy in select cases.
CT	Cross-sectional imaging for parenchymal/duct morphology, excludes complications.	Good for advanced disease; detects calcifications/complications.	Poor sensitivity in early stage; radiation exposure; low soft tissue contrast.	Useful for suspicion of complications or when other modalities unavailable.
MRI/MRCP	Visualizes ductal anatomy, parenchyma, and fluid collections; secretin-enhanced MRCP (sMRCP) increases functional assessment.	High soft tissue contrast; secretin reveals ductal function.	Availability/cost issues; contraindications in metal implants; interpretation variability.	Excellent non-invasive option for both structure and function assessment.
MRI Elastography	Quantifies tissue stiffness to indicate early fibrosis.	Non-invasive, quantitative fibrosis measurement.	Emerging technique; needs standardization, limited access.	May become adjunct to MRI in fibrosis quantification.

Abbreviations: EUS—Endoscopic Ultrasound; EUS-nCLE—Endoscopic Ultrasound-guided Needle-based Confocal Laser Endomicroscopy; FNA—Fine Needle Aspiration; nCLE—Needle-based Confocal Laser Endomicroscopy; sMRCP—Secretin-enhanced Magnetic Resonance Cholangiopancreatography.

## Data Availability

No new data were created or analyzed in this study. Data sharing is not applicable to this article.

## Abbreviations

The following abbreviations are used in this manuscript:

AI	Artificial Intelligence
AIP	Autoimmune Pancreatitis
CFTR	Cystic Fibrosis Transmembrane Conductance Regulator
CP	Chronic Pancreatitis
CT	Computed Tomography
CTSI	Computed Tomography Severity Index
ECP	Early Chronic Pancreatitis
EUS	Endoscopic Ultrasound
ERCP	Endoscopic Retrograde Cholangiopancreatography
GWAS	Genome-Wide Association Studies
IL-6	Interleukin-6
JPS	Japanese Pancreas Society
MRI	Magnetic Resonance Imaging
MRCP	Magnetic Resonance Cholangiopancreatography
NGS	Next-Generation Sequencing
PERT	Pancreatic Enzyme Replacement Therapy
PRSS1	Protease, Serine, 1 (cationic trypsinogen gene)
RAP	Recurrent Acute Pancreatitis
sCD163	Soluble CD163 (macrophage activation marker)
SPINK1	Serine Protease Inhibitor Kazal Type 1
TNF-α	Tumor Necrosis Factor-alpha
